# Emerging Therapeutic Innovations for Vitiligo Treatment

**DOI:** 10.3390/cimb47030191

**Published:** 2025-03-14

**Authors:** Weiran Li, Penghao Dong, Guiyuan Zhang, Junjie Hu, Sen Yang

**Affiliations:** 1 Institute of Dermatology and Department of Dermatology, The First Affiliated Hospital, Anhui Medical University, Hefei 230000, China; dph511@163.com (P.D.); yxdlao@sina.com (G.Z.); 18961566781@163.com (J.H.); 2 Department of Dermatology, Dushu Lake Hospital Affiliated to Soochow University, Suzhou 215128, China; 3 Key Laboratory of Dermatology, Ministry of Education of the People’s Republic of China, Hefei 230000, China

**Keywords:** vitiligo, pathogenesis, emerging drugs, JAK inhibitors, immunotherapy

## Abstract

Vitiligo is a chronic autoimmune disorder with a multifactorial etiology, typically manifesting as localized or generalized hypopigmentation or depigmentation of the skin and mucous membranes. The pathogenesis of vitiligo is complex and significantly impacts patients’ quality of life. Although traditional treatments such as hormone therapy, topical medications, and laser therapy can help control the disease to some extent, their outcomes remain unsatisfactory. Therefore, ongoing research is crucial to explore and develop novel treatment strategies while assessing their efficacy and safety. This review aims to classify and summarize various new candidate drugs for vitiligo currently undergoing clinical trials, providing a reference for clinical practice. Recent advancements in the understanding of the pathogenesis of vitiligo have facilitated the development of potential treatment strategies, such as Janus kinase inhibitors, cytokine blockers, and agents targeting tissue-resident memory or regulatory T cells. These emerging therapies offer hope to patients with vitiligo, though further investigation is needed to confirm their safety, efficacy, and optimal treatment regimens.

## 1. Introduction

Vitiligo is a chronic acquired autoimmune skin disorder characterized by the destruction of melanocytes, leading to the appearance of sharply demarcated, white, non-scaly macules and patches surrounded by normal skin [1]. The prevalence of vitiligo varies among studies but is estimated to affect approximately 0.5 to 2.0% of the global adult and pediatric populations, with no significant variation in incidence across sexes, races, or geographical regions [2]. Individuals with vitiligo often face discrimination, and psychosocial impacts, including anxiety and stigmatization, can significantly affect their quality of life, particularly among those with darker skin tones [3]. Many patients may experience behavioral changes, anxiety disorders, social phobia, sleep disturbances, and cognitive and emotional impairments [4]. Approximately half of all vitiligo cases manifest before the age of 20 years, with nearly 80% occurring before the age of 30 years, making these psychosocial impacts particularly alarming [5]. Moreover, the chronic nature of the disease and its high recurrence rate often necessitate long-term treatment strategies [6].

The hallmark of vitiligo is loss of epidermal melanocytes, primarily attributed to the activity of autoreactive CD8+ cytotoxic T cells. Research into the underlying causes of this condition is ongoing [7]. Current evidence suggests that a variety of mechanisms, including genetic predisposition, oxidative stress, neural factors, melanocyte destruction, and autoimmune processes, may contribute either independently or synergistically. Although numerous theories have been proposed, the principal pathogenic mechanisms can be categorized into two main pathways: those mediating autoimmunity and those affecting melanocyte homeostasis [8,9]. The autoimmunity pathway involves the Janus kinase (JAK)/signal transducer and activator of transcription (STAT) pathway, the heat shock protein (HSP) 70-plasmacytoid dendritic cell interferon (IFN)-α-C-X-C motif chemokine ligand (CXCL)9/10 axis, and the regulatory functions of regulatory T cells (Tregs) and tissue-resident memory T cells (TRMs). Conversely, the melanocyte homeostasis pathway encompasses mechanisms such as the unfolded protein response system triggered by endoplasmic reticulum stress, the nuclear factor erythroid 2-related factor 2-antioxidant responsive element antioxidant axis, the corticotropin-releasing hormone-proopiomelanocortin axis, and the Wnt/β-catenin signaling pathway.

Autoimmune diseases comprise a diverse set of conditions with intricate pathologies, typically resulting from a confluence of genetic susceptibility and environmental co-factors. Historically, due to the limited understanding of the underlying molecular mechanisms of autoimmune diseases, the conventional treatment of autoimmune disorders such as systemic lupus erythematosus (SLE), multiple sclerosis, type 1 diabetes mellitus (T1DM), psoriatic arthritis, and rheumatoid arthritis has relied on a broad spectrum of (largely untargeted) immunosuppressive agents. Specifically, glucocorticosteroids (e.g., prednisone, dexamethasone, and hydrocortisone) and non-steroidal anti-inflammatory drugs (NSAIDs; e.g., ibuprofen and naproxen) have been widely used to impede the assault of autoreactive immune cells on host tissues.

Analogously, the primary objective in treating vitiligo, a prevalent autoimmune skin disease, is to suppress autoimmune-mediated damage and stimulate melanin synthesis by the remaining intact peripheral melanocytes. A systematic review in 2015 systematically described the traditional treatment of vitiligo [2]. Topical corticosteroids, such as clobetasol propionate, remain a first-line treatment for localized disease due to their immunosuppressive effects, which may arrest melanocyte destruction and promote repigmentation. However, long-term use is associated with cutaneous adverse effects, including atrophy and telangiectasia. Phototherapy modalities, particularly psoralen plus ultraviolet A (PUVA) and narrowband ultraviolet B (NB-UVB), have been widely investigated. PUVA, involving oral or topical psoralens followed by UVA exposure, demonstrates efficacy in darker skin types but carries risks of nausea and phototoxicity. NB-UVB monotherapy or combination regimens with vitamin D analogues (e.g., calcipotriol) or corticosteroids have emerged as safer alternatives, with improved repigmentation outcomes in randomized controlled trials. Immunomodulatory agents, including tacrolimus and pimecrolimus, offer non-steroidal alternatives for sensitive areas like the face, though transient burning sensations are common. Surgical interventions, such as autologous skin grafting or melanocyte transplantation, are reserved for stable disease but are limited by technical complexity and scarring risks. Oral therapies, including ginkgo biloba and antioxidant combinations, show modest benefits in halting progression or enhancing repigmentation when paired with phototherapy. In the contemporary era, as our understanding of the pathogenesis of autoimmune skin diseases, including vitiligo, at the molecular level has deepened, novel targeted therapies have emerged. These therapies are designed to augment the therapeutic effectiveness of autoimmune skin disease treatment while minimizing adverse effects.

The JAK/STAT pathway is one of the most closely studied targets in current vitiligo treatment research [10]. Recently, the United States Food and Drug Administration (U.S. FDA) approved ruxolitinib cream, the first topical JAK inhibitor specifically for vitiligo treatment [11,12]. Such medications hold the potential to replace oral immunosuppressants and corticosteroids, which are associated with nonspecific, broad immunosuppressive effects and a higher risk of side effects.

As our understanding of vitiligo pathogenesis expands beyond the JAK/STAT pathway, we are entering a new era in vitiligo treatment. The treatment paradigm is shifting with innovative drugs entering or nearing clinical trials. This review summarizes the prospects and challenges of these emerging therapies, offering valuable insights for improving the clinical management of vitiligo.

## 2. Literature Search Strategy

In December 2024, the PubMed database was searched for this study. Broad keyword expansion was employed using the search terms: (vitiligo) AND (JAK inhibitor OR melanocyte OR JAK/STAT-1 signaling pathway OR drug OR emerging drug OR targeted drug OR biologic OR therapy OR emerging therapy OR targeted therapy OR randomized controlled trial OR case report). Once the search was completed, the researchers evaluated the titles and abstracts of the retrieved literature to determine their relevance. The inclusion criteria were as follows: (1) the studies were written in English, and (2) they focused on the efficacy, tolerability, and adverse effects of approved, supra-indicated, and investigational treatments for vitiligo. Systematic reviews, narrative reviews, guidelines, drafts, and conference abstracts were excluded. Subsequently, the researchers reviewed the full-text of the literature that met the inclusion criteria. Additionally, systematic and narrative reviews on similar topics were examined, and their reference lists were manually screened.

## 3. JAK Inhibitors

### 3.1. JAK-STAT Signaling Pathway

**Figure 1:**
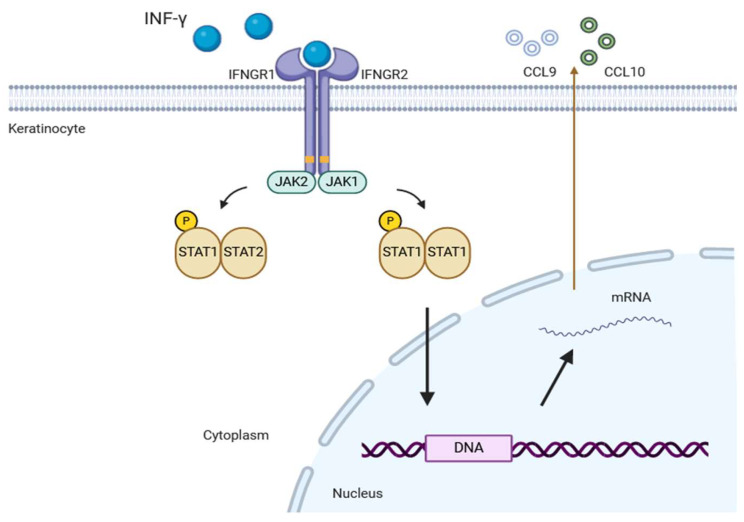
The role of IFN-γ signaling and the Janus kinase (JAK)/signal transducer and activator of transcription (STAT) pathway in vitiligo.

JAK is a multi-domain non-receptor tyrosine kinase that plays a key role in cell signal transduction by activating the STAT signaling pathway, particularly in T cell differentiation and immune regulation [13]. The human JAK family comprises JAK1, JAK2, JAK3, and tyrosine kinase 2 (TYK2), all of which are essential for transmitting extracellular signals from various cytokines [14]. JAKs associate with the cytoplasmic domains of type I and type II chemokine receptors, and their ligands include IFN-γ, among others. IFN-γ is a key cytokine produced by CD8+ T cells and plays a central role in the pathogenesis of the disease. When cytokines bind extracellularly to their receptors, JAKs are brought into proximity, leading to autophosphorylation and self-activation. Activated JAKs phosphorylate STATs, triggering STAT dimerization, nuclear translocation, DNA binding, and the regulation of gene expression (Figure 1) [15].

**Figure 2:**
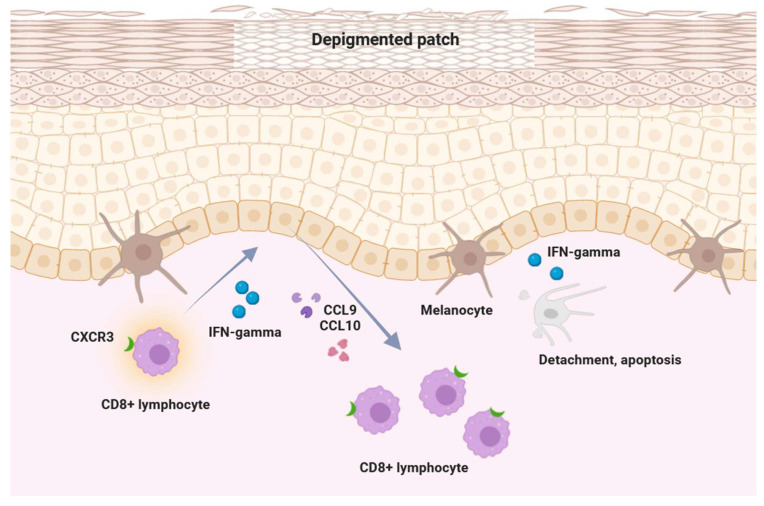
Elucidation of the pathogenesis of vitiligo: The IFN-γ—chemokine axis, along with its associated positive feedback loop.

Available data suggest that in the pathogenesis of vitiligo, the expression of JAK1 and JAK3 is upregulated, and key cytokines exert their effects via JAK receptors [16]. IFN-γ can activate JAK1 and JAK2 dimers, subsequently stimulating keratinocytes to express CXCL9 and CXCL10. This process further increases the recruitment of CD8+ T cells targeting melanocytes (Figure 2) [17]. Given that most cytokines implicated in the pathogenesis of vitiligo signal through the JAK pathway, JAK inhibitors represent a potential therapeutic strategy to halt disease progression.

As of July 2022, ruxolitinib has been the only JAK inhibitor approved by the U.S. FDA for treating non-segmental vitiligo in patients aged 12 years and older [18]. Other JAK inhibitors, such as baricitinib, tofacitinib, and ritlecitinib, are currently undergoing clinical trials (Table 1).

### 3.2. Ruxolitinib

Ruxolitinib (INCB-018424) is a small-molecule inhibitor that selectively targets JAK1 and JAK2 [20]. Initially developed for treating polycythemia vera and primary myelofibrosis, its potential in treating vitiligo was identified when rapid skin repigmentation was observed in male patients with vitiligo and alopecia areata receiving oral ruxolitinib. This effect was accompanied by a significant decrease in serum CXCL10 levels, suggesting that the mechanism of action of ruxolitinib may involve the blockade of IFN-γ signaling and JAK inhibition [21]. Furthermore, studies using mouse and human tissues have shown that ruxolitinib inhibits the differentiation and migration of human dendritic cells (DCs) in vitro. In vivo, it was shown to reduce DC-induced antigen-specific CD4+ and CD8+ T cell responses, as well as the induction of CD8+ cytotoxic T cell responses, which are key cellular responses involved in the pathogenesis of vitiligo [22]. A recent ongoing phase 2 study (NCT04896385), currently in the recruitment stage, aims to investigating the mechanism of action of ruxolitinib cream for the treatment of vitiligo by evaluating the changes in immune biomarkers, including CXCL10.

Based on preliminary findings in mouse and human tissues, clinical trials of topical ruxolitinib were initiated. In a 20-week open-label phase 2 trial (NCT02809976), 12 patients applied 1.5% ruxolitinib cream to vitiligo lesions twice daily. By the end of the trial, significant improvement in facial lesions was observed in four patients, with an average Vitiligo Area Scoring Index (VASI) reduction of 23% across all participants [23]. A subsequent 32-week extension study was conducted, though no improvement was observed in non-responsive skin lesions. Interestingly, five patients maintained their treatment response for up to 6 months after drug discontinuation during the follow-up period [24].

In another double-blind phase 2 trial (NCT03099304), 157 adult patients with vitiligo from 26 hospitals in the U.S. were randomized in a 1:1:1:1:1 ratio to receive one of the following topical application treatments for 24 weeks: 1.5% ruxolitinib cream twice daily, 1.5% once daily, 0.5% once daily, 0.15% once daily, or vehicle cream [12]. The primary endpoint was the percentage of patients achieving > 50% improvement in facial VASI (F-VASI50) at week 24 compared to baseline. At the end of the 24-week treatment period, significantly more patients in the 1.5% ruxolitinib cream twice daily, 1.5% ruxolitinib cream once daily, and 0.5% ruxolitinib cream once daily achieved F-VASI50 compared to the control group. These three responsive groups continued their respective treatment regimens through week 52. By the study end, patients in all three treatment groups showed significant repigmentation of vitiligo lesions and good dose tolerance. These findings highlight the potential of topical ruxolitinib as a promising therapy for vitiligo treatment. However, the study had certain limitation. The average age of the patients was >40 years, and only patients with non-segmental vitiligo were included. In addition, in both studies, the average overall affected body surface area of affected by vitiligo among the enrolled patients was <10%, and the maximum topical exposure to ruxolitinib was limited to 3.74 g.

Despite the limited response observed in some patients following treatment with ruxolitinib cream, the latest findings from the TRuE-V long-term extension study (weeks 52–104) provide promising insights. Among patients who exhibited zero or minimal (<25%) repigmentation at week 24, a significant proportion demonstrated notable improvement at week 104. Specifically, 54.9% (39 out of 71) achieved F-VASI75, and 50.0% (53 out of 106) reached T-VASI50. These findings emphasize the therapeutic benefits of prolonged use of ruxolitinib cream.

Moreover, combining ruxolitinib cream with phototherapy has shown potential to enhance repigmentation, especially in patients with suboptimal responses to ruxolitinib alone. Improved outcomes and good tolerance with combination therapy have been observed, highlighting its significant application potential in clinical practice. Unlike the controlled settings of clinical trials, many physicians explore tailored treatment regimens in routine practice, making combination therapy a valuable approach. Rosmarin et al. initiated a clinical trial (NCT05247489) investigating the combined use of ruxolitinib cream and phototherapy; while the study has concluded, its results remain unpublished. Additionally, an ongoing open-label phase 2 study is investigating the use of ruxolitinib cream in patients with genital vitiligo (NCT05750823).

### 3.3. Tofacitinib

Tofacitinib is a JAK1 and JAK3 inhibitor with partial inhibitory effects on JAK2 and TYK2. It is approved for the oral treatment of moderate-to-severe rheumatoid arthritis [25]. Oral and topical formulations of tofacitinib have demonstrated efficacy in treating immune-mediated skin conditions, including plaque psoriasis and atopic dermatitis [26,27].

A case report described a female patient with refractory vitiligo covering 10% of the body surface area who responded well to oral tofacitinib. After 5 months of treatment, complete repigmentation was achieved on the forehead and hands, with only 5% of the total skin area remaining unresponsive. No adverse effects were reported during the treatment period [28]. In a retrospective study of 10 patients with vitiligo treated with tofacitinib at doses of 5–10 mg/day or twice daily for an average of 9.9 months, half of the patients experienced repigmentation in sun-exposed areas or areas receiving phototherapy alone. Tofacitinib reduced T cell infiltration and chemokine expression in responsive and non-responsive skin lesions, suggesting that phototherapy might be essential for regenerating melanocytes [29]. This is consistent with the finding that sun-exposed areas, such as the hands and face, show a better response to topical ruxolitinib treatment [23].

However, owing to safety warnings issued by the FDA regarding systemic JAK inhibitors, particularly tofacitinib, which has been associate with increased risk of serious cardiovascular events, topical formulations are preferred. In another study, 16 patients with vitiligo, including 11 with generalized vitiligo, used 2% tofacitinib topical cream. Consistent with previous studies, patients with facial lesions and darker skin tones had a more significant response, whereas no enhanced response was observed in patients receiving phototherapy concurrently, contrasting with earlier reports [19]. The exploration of tofacitinib remains in its early stages, with ongoing phase I trials (NCT05293119). Further research is needed to establish its safety and efficacy, as well as to clarify the role of combination therapy with phototherapy.

### 3.4. Baricitinib

Baricitinib is a selective JAK1 and JAK2 inhibitor. A case report described the successful use of baricitinib for repigmentation of vitiligo lesions [25]. After switching from tofacitinib to baricitinib, complete repigmentation was achieved on the forearm and hands, suggesting the potential of baricitinib as a treatment for vitiligo. Clinical studies on baricitinib are limited. However, one study reported that four patients achieved repigmentation rates ranging from 59.29% to 74.18% after 12 weeks of oral baricitinib treatment, without significant side effects [30]. Moreover, in vitro studies have demonstrated that baricitinib promotes tyrosinase (TYR) activity, TYR and tyrosinase-related protein-1 gene expression, and melanin synthesis in cultured melanocytes [31]. Given that IFN-γ signaling is mediated through JAK1/2, baricitinib may be particularly effective in treating vitiligo. This is evidenced by its good safety profile in atopic dermatitis and alopecia areata treatments [32]. Seneschal et al. [33] conducted a multicenter, double-blind trial assessing baricitinib (4 mg/day) plus NB-UVB in adults with severe, active non-segmental vitiligo (NCT04822584). Thirty-seven patients received baricitinib and twelve received a placebo for 36 weeks, with NB-UVB added from week 12. At week 36, baricitinib showed a −44.8% mean reduction in VASI vs. −9.2% for placebo, not surpassing the prespecified 42.9% threshold. Post hoc analysis revealed significant between-group differences, greater clinical/QOL improvements with baricitinib, and comparable adverse events. This study supports baricitinib-NB-UVB as a potential therapy for active vitiligo.

### 3.5. Upadacitinib

Upadacitinib is a selective JAK1 inhibitor commonly used for treatment of rheumatic diseases and atopic dermatitis. A case report described a 16-year-old boy with vitiligo and atopic dermatitis who showed significant improvement in facial and neck repigmentation after receiving oral upadacitinib, especially in sun-exposed areas [34]. In a recent study involving twelve patients with refractory vitiligo, seven patients achieved an average pigmentation improvement rate of 51.4%, and the treatment effect was more pronounced on facial lesions compared to acral lesions [35].

A recent phase 2 study further explored the impact of upadacitinib on the quality of life in patients with non-segmental vitiligo (NCT04927975). Treatment with a 22 mg dose of upadacitinib led to a greater proportion of patients reporting less conspicuous vitiligo lesions compared to the placebo group (11.6% vs. 0%; *p* < 0.05). Additionally, the study exhibited significant reduction in Dermatology Life Quality Index scores for upadacitinib-treated patients relative to the placebo group (−2.2 vs. −0.6; *p* < 0.05). Currently, a phase 3 study evaluating the efficacy and safety of upadacitinib for the treatment of non-segmental vitiligo is ongoing (NCT06118411).

### 3.6. Ritlecitinib

Ritlecitinib is a kinase inhibitor that irreversibly targets JAK3 and other tyrosine kinase family members, with potential implications for treating immune-mediated diseases such as hepatocellular carcinoma [36]. Owing to its potent inhibition of interleukin (IL)-15 signaling, ritlecitinib was recently approved in the U. S. and Japan for the treatment of severe alopecia areata [37]. In alopecia areata, IL-15 signaling stimulates the survival of CD8+ memory T cells, promotes the proliferation and maintenance of T cells and natural killer (NK) cells, and induces IFN-γ production by CD8+ T cells. This mechanism is similar to the pathogenesis of vitiligo, where IL-2 and IL-15 activate TRM cells, leading to the secretion of perforin, granzyme B, and IFN-γ, which are cytotoxic to melanocytes [38,39]. Furthermore, TRM cells produce CXCL9 and CXCL10, which interact with CXCR3 on TRM cells, facilitating their recruitment to the skin [40]. Therefore, ritlecitinib shows promise in inhibiting the downstream signaling of IL-2 and IL-15 and reducing the cytolytic functions of CD8+ T cells and NK cells.

Ezzedine et al. conducted a phase 2b clinical trial demonstrating that oral ritlecitinib significantly improved repigmentation in patients with active non-segmental vitiligo [36]. A total of 364 patients were randomized into six groups, including five treatment groups and one placebo group. Two groups received a loading dose of 200 or 100 mg/day for the first four weeks, followed by a maintenance dose of 50 mg/day for the subsequent 20 weeks. The other three groups received daily doses of 50 mg, 30 mg, or 10 mg for 24 weeks without a loading dose. Results showed the greatest improvement in the F-VASI at week 24 in the group receiving 50 mg/day group with a 200 mg loading dose (−21.2%), followed by the 50 mg/day and 30 mg/day groups without a loading dose (−18.5 and −14.6%, respectively). All treatment groups showed statistically significant improvements compared with the placebo group. The highest percentage of patients achieving meaningful F-VASI improvement at week 24 (12.1%) was observed in the 50 mg/day group with 200 mg loading dose. After 24 weeks, an extension study demonstrated continued improvements in F-VASI and the Total-VASI (T-VASI), supporting the effectiveness of long-term treatment. Interestingly, the 30 mg/day group reported high patient satisfaction and a significant decrease in T-VASI at week 48, suggesting the need for further exploration of optimal treatment dosing strategies. The main limitations of this study include the exclusion of patients with stable vitiligo and the predominantly white study population. A phase 3 clinical trial (NCT05583526) is currently underway to evaluate the efficacy and safety of ritlecitinib (50 mg/day) in patients with stable and active non-segmental vitiligo.

### 3.7. Other JAK Inhibitors

With the expanding potential indications for JAK inhibitors and growing evidence of their efficacy, the registration of clinical trials for new JAK inhibitors continues to rise.

Efinetinib (ATI-50002) is a dual JAK1 and JAK3 inhibitor [1]. A recent open-label phase 2 study (NCT03468855) evaluated the safety and efficacy of a 0.46% efinetinib solution for the treatment of non-segmental facial vitiligo. Among the 34 enrolled patients, 23 completed the 24-week treatment regimen with twice-daily application of efinetinib solution. The results reported improvements in the F-VASI and the Vitiligo Noticeability Scale, suggesting its therapeutic potential.

Povorcitinib (INCB054707) is a selective JAK1 inhibitor, primarily used for hidradenitis suppurativa [41]. Phase 3 clinical trials are currently underway to evaluate the efficacy and safety of povorcitinib in patients with non-segmental vitiligo (NCT06113445, NCT06113471). In a previous phase 2 study (NCT04818346), 171 patients with non-segmental vitiligo were randomized to receive 15, 45, or 75 mg povorcitinib or placebo for 24 weeks. During the subsequent 28-week extension period, patients continued to receive either 45 or 75 mg doses of povorcitinib. The primary endpoint was the percentage change in the T-VASI from baseline to week 24.

Cerdulatinib (PRT062070) is a spleen tyrosine kinase/JAK dual kinase inhibitor. In a phase 2a randomized, double-blind, controlled study (NCT04103060), the safety and tolerability of 0.37% cerdulatinib gel applied twice daily for topical treatment of vitiligo are being evaluated.

## 4. STAT Inhibitors

Simvastatin is a potent lipid-lowering agent with immunomodulatory properties, commonly used in the treatment of dyslipidemia and cardiovascular diseases. Evidence suggests that simvastatin may act as an immunomodulator in vitiligo and potentially prevent associated metabolic complications [42]. Agarwal et al. [43] demonstrated that simvastatin blocks IFN-γ signaling by inhibiting STAT1 activation. Moreover, in a vitiligo mouse model, simvastatin decreased the number of CD8+ T cells in leukoderma lesions and prevented and reversed depigmentation.

A case report indicated that the oral administration of simvastatin in a hyperlipidemic patient with vitiligo led to varying degrees of repigmentation [44]. Additionally, Shaker et al. [45] observed significant benefits in a cohort of 79 non-segmental patients with vitiligo with dyslipidemia who took 80 mg of simvastatin orally daily until blood lipid levels normalized or for a treatment period of 4 months. The results showed a significant positive correlation between the VIDA score and serum total cholesterol and low-density lipoprotein levels. This suggests that simvastatin significantly reduces the VIDA score while improving blood lipid levels. In another study, Zhang et al. [46] treated five patients with vitiligo with a combination of simvastatin and 0.1% topical tacrolimus ointment. Three patients showed remarkable efficacy without severe adverse reactions, suggesting potential synergistic benefits. However, a randomized, double-blind, placebo-controlled phase II clinical trial (NCT01517893) conducted by Vanderweil et al. [47] yielded less promising results. Patients with vitiligo received 40 mg of simvastatin orally daily for the first month, followed by 80 mg daily for the following 5 months (treatment group) or a placebo (placebo group). After six months of treatment, the treatment group experienced a 26% increase in the average VASI score, compared with no significant change in the placebo group. Additionally, the treatment group experienced adverse reactions such as self-limiting myalgia and diarrhea. This study implies that oral simvastatin at 80 mg daily failed to achieve satisfactory efficacy for vitiligo treatment. Discrepancies observed in the results of several extant studies can likely be ascribed to limitations regarding potential toxicity at human doses when compared to animal studies. Moreover, variations in efficacy may be closely associated with factors such as the variable disease duration among subjects, small sample sizes, or the absence of sensitive measures for treatment response.

Since high-dose simvastatin is required for systemic use, potential toxicity may limit its therapeutic effects in patients with vitiligo. This limitation underscores the need for alternative strategies to harness simvastatin’s benefits while minimizing systemic risks. Oxidative stress is strongly associated with the occurrence and progression of vitiligo. Studies have shown that simvastatin can directly act on melanocytes, activating key intracellular antioxidant pathways and protecting against oxidative stress-induced damage, which is an entirely distinct mechanism from its immunomodulatory effects in vitiligo treatment [48]. This finding suggests the potential for using simvastatin as a topical treatment for vitiligo. Considering that topical therapy can achieve sufficiently high local concentrations without inducing systemic toxicity, the topical application of statins and the combination of topical therapy with other treatment modalities hold promise. For instance, trials have explored the use of topical atorvastatin or simvastatin in conjunction with narrowband UVB (NB-UVB) phototherapy, yielding promising preliminary outcomes [49,50]. Nevertheless, additional clinical evidence is required to validate their efficacy.

## 5. Cytokines

A IFN-α is a cytokine that plays a crucial role in inflammatory, infectious, and autoimmune diseases. In the context of autoimmune diseases such as vitiligo and cutaneous lupus erythematosus, the HSP 70–plasmacytoid dendritic cell–IFN-α–CXCL9/10 axis has been proposed as a key pathogenic mechanism [51,52]. Anifrolumab, a human monoclonal antibody targeting subunit 1 of the type I IFN receptor, has been approved by the FDA for the treatment of moderate-to-severe systemic lupus erythematosus [53]. Currently, a phase 2 clinical trial is underway to evaluate the efficacy and tolerability of anifrolumab combined with phototherapy in adult patients with progressive vitiligo compared to phototherapy alone (NCT05917561).

Tumor necrosis factor α (TNF-α) is an important cytokine primarily secreted by macrophages and monocytes, playing a central role in inflammation, apoptosis, and immune system development. In a series of case reports on the treatment of generalized vitiligo vulgaris, two patients received a subcutaneous injection of adalimumab, three received intravenous infliximab, and seven were treated with subcutaneous injection of etanercept [52,53,54,55]. Among these, only infliximab and etanercept showed potential efficacy in two reports. In the remaining cases, no exacerbation of the depigmented areas was observed, but pigment repigmentation occurred either. Therefore, the efficacy of anti-TNF-α drugs in the treatment of vitiligo remains unconfirmed, necessitating larger-scale and long-term studies.

Tregs play a key role in maintaining self-tolerance by suppressing autoreactive effector T cell activity. Dysfunctional Tregs are a potential therapeutic targets in vitiligo [56]. IL-2 is essential for the survival and function of Tregs in peripheral tissues [57]. Low-dose IL-2 has been shown to induce Treg expansion, thereby maintaining immune homeostasis. In preclinical studies, MK-6194 (PT101), an IL-2 mutant Fc fusion protein, has shown promise in activating and expanding Tregs in humanized NSG mice and nonhuman primates [58]. This expansion enhances the function and stability of Tregs by upregulating the expression of forkhead box protein P3 and CD25, presenting a therapeutic role in vitiligo. Currently, a phase 2 clinical study (NCT06113328) is underway to evaluate the efficacy, safety, and tolerability of MK-6194 in patients with non-segmental vitiligo.

IL-15 is critical in the regulation of TRM signaling TRM cells contribute to disease maintenance and recurrence by promoting the recruitment and proliferation of cytotoxic CD8+ T cells [59]. Studies have found that elevated levels of IL-15 in the serum of patients with vitiligo correlate with disease severity [60]. This makes IL-15 a potential target for the treatment of patients with vitiligo. AMG714, a fully human immunoglobulin monoclonal antibody, binds to IL-15 and directly blocks its T cell activation and proliferation signals [61]. A phase 2 clinical trial (NCT04338581) is in progress to evaluate the efficacy of AMG714 in active or stable non-segmental vitiligo.

## 6. Immune Checkpoints

Abatacept, an immunoglobulin G1 fusion protein, is approved for the treatment of moderate-to-severe rheumatoid arthritis. This drug binds to the extracellular domain of cytotoxic T-lymphocyte-associated antigen-4 (CTLA-4) through its Fc region, thereby negatively regulating T cell activation [62,63]. In patients with vitiligo, the levels of soluble CTLA4 and full-length CTLA4 mRNA are decreased [56]. Furthermore, the downregulation of CTLA-4 expression in regulatory T cells weakens their inhibitory effect on autoreactive CD8+ T cells [64]. Currently, a phase 1 trial (NCT02281058) is underway to verify the potential therapeutic benefits of abatacept for vitiligo.

## 7. T-Cell Metabolism

Considering the significant role of oxidative stress in the pathogenesis of vitiligo and the defective autophagy observed in patients with vitiligo, promoting autophagy has emerged as a promising treatment option [65].

Since the mammalian target of rapamycin (mTOR) functions as an inhibitory factor of autophagy, exploring the potential of rapamycin, which suppresses mTOR activity, is of great interest [66]. An in vitro study demonstrated that rapamycin treatment led to the elevated expression of microphthalmia-associated transcription factor, TYR, and microphthalmia-associated transcription factor TYRP1/2 in melanocytes [67]. Additionally, the mTOR pathway is implicated in the decline of Tregs, as evidenced in lupus erythematosus [68]. In a study involving h3TA2 mice, rapamycin enhanced the number of Tregs, leading to improvement in progressive or established depigmentation [69]. The effectiveness and tolerability of rapamycin in the treatment of non-segmental vitiligo are currently being evaluated in a clinical trial (NCT05342519) using a daily application of 0.1 or 0.001% cream to lesions occupying less than 2% of the body surface area.

Over the past few decades, metformin, a staple treatment for type 2 diabetes, has been well known for its diverse functions, including antiproliferative and antioxidant capabilities [70]. Metformin activates the 5′-AMP-activated protein kinase (AMPK) pathway and promotes mitochondrial respiration and oxidation in anti-inflammatory cells, such as Tregs and M2 macrophages, while limiting the glycolytic capacity of pro-inflammatory neutrophils, M1 macrophages, and effector T cells [71,72]. Furthermore, studies have revealed that metformin can reduce mTOR signaling via AMPK-dependent and AMPK-independent mechanisms, resulting in an increase in Tregs [70]. As an inhibitor of mitochondrial metabolism, metformin can restore the normal metabolism of T cells and diminish the production of IFN-γ [73]. These multifaceted effects suggest its potential as a novel addition to existing therapeutic approach for vitiligo. Currently, a phase 2 clinical trial is in progress to investigate the efficacy and safety of metformin for the management of vitiligo (NCT05607316).

## 8. α-Melanocyte-Stimulating Hormone

Afamelanotide, an analog of α-melanocyte-stimulating hormone (α-MSH), is primarily used for treating patients with erythropoietic protoporphyria [74]. It binds to the melanocortin 1 receptor, activating downstream signaling pathways related to melanogenesis and anti-inflammation in melanocytes, keratinocytes, endothelial cells, fibroblasts, and mast cells [75,76]. In recent years, several preliminary studies have evaluated the efficacy and safety of afamelanotide combined with phototherapy for the repigmentation of non-segmental vitiligo [77].

In a recent randomized trial for the treatment of non-segmental vitiligo, the combination of afamelanotide and phototherapy achieved a higher rate of repigmentation (48.64%) compared to NBUVB monotherapy (33.26%) [78]. An open-label study further demonstrated the efficacy and tolerability of afamelanotide [79]. In these studies, the most common side effect observed was hyperpigmentation, with occasional reports of headaches, dizziness, and nausea. Although afamelanotide stimulates the melanocortin 1 receptor in epidermal melanocytes to increase their pigmentation and proliferation, it does not induce stem cells to regenerate into melanocytes [80]. Currently, a phase 2 clinical study is underway to evaluate the effects of afamelanotide implants alone (NCT05210582). In addition, a phase 3 clinical trial is underway to compare the efficacy of afamelanotide combined with NBUVB phototherapy vs. NBUVB monotherapy (NCT06109649).

## 9. Phosphodiesterase-4

Phosphodiesterase-4 (PDE-4) is an intracellular enzyme that degrades cyclic adenosine monophosphate (cAMP) into 5′-adenosine monophosphate [81]. PDE-4 inhibitors prevent the hydrolysis of cAMP, thereby increasing intracellular cAMP levels. This mechanism inhibits the production of IL-17, IL-23, TNF-α, and IFN-γ, while promoting IL-10 production. Overall, these compounds play a protective role against vitiligo through their antioxidant, anti-inflammatory, and melanogenesis-promoting effects [82,83].

Currently, evidence supporting the use of PDE-4 inhibitors in the treatment of vitiligo is limited to case reports involving oral apremilast and topical crisaborole. A case report on oral apremilast demonstrated its potential in the treatment of vitiligo [84]. Two clinical trials investigated the addition of apremilast to NB-UVB treatment, but their findings were contradictory [85,86]. Recently, Sharma et al. [87] reported that the inclusion of apremilast to standard treatment reduced the progression of vitiligo and accelerated the clinical improvement of the disease.

Evidence for topical PDE-4 inhibitors, such as crisaborole, is even more limited, with only a few case reports supporting its use [88,89]. A phase 2 study (NCT05298033) is underway to evaluate the combination of 2% crisaborole ointment and 0.01% PF-07038124 (a PDE-4 inhibitor) ointment with or without phototherapy.

## 10. 5-Fluorouracil

5-Fluorouracil (5-FU) is a widely used systemic chemotherapeutic drug for cancer treatment. Its topical formulations are convenient to use, exhibit good efficacy, and have minimal adverse effects. In recent years, 5-FU has gained attention as a treatment for vitiligo. Topical and intradermal formulations are usually combined with techniques such as manual and motorized dermabrasion, acupuncture, and carbon dioxide fractional lasers to enhance efficacy. Observational [90,91,92] and experimental studies have compared the outcomes of various combinations involving 5-FU. For example, combined use of 5-FU cream and microneedling demonstrated superior repigmentation rates compared to microneedling alone [93,94], 5-FU alone [95], and the combined application of microneedling with tacrolimus [96,97] or 308 nm excimer light [98]. The overall qualitative response of plaques treated with the combination of 5-FU and microneedling was better, and there was a statistically significant advantage in the repigmentation rate compared to tacrolimus or microneedling alone. All studies showed a significantly higher response rate (repigmentation > 75%) and a reduced rate of poor responses (<25%). In a study by Saad et al. [98], combined treatment of microneedling followed by the application of 5-FU and excimer light showed significant effect to excimer light alone. To promote the penetration of 5-FU, techniques such as dermabrasion similar to microneedling can be utilized [99,100]. However, common side effects of these two techniques were erythema and pruritus.

Recent studies also tested intradermal injections of 5-FU (50 mg/mL) every two weeks and compared its effect with that of triamcinolone acetonide (3 mg/mL) injections at the same frequency [101,102]. Intradermal injection of 5-FU showed the superior overall improvement compared to triamcinolone acetonide. In addition, a combination of 5-FU and phototherapy [102] showed a better effect than phototherapy alone. However, intradermal injections are associated with higher rates of side effects. Most patients reported pain and burning sensation during the injection, as along with blister and ulcer formation.

## 11. Trichloroacetic Acid

The repigmentation mechanism of Trichloroacetic acid (TCA) in vitiligo is likely associated with its capacity to trigger inflammation and subsequent post-inflammatory hyperpigmentation [103]. Moreover, TCA-induced necrosis and the ensuing trauma may also stimulate melanocyte proliferation through the production of pro-opiomelanocortin and melanocortin, along with the release of growth factors and inflammatory mediators [104].

Nofal et al. [103] conducted a study involving 100 patients and reported an 80% response rate for eyelid vitiligo. The response rates were lower on the face, torso, and extremities when variable concentrations of TCA were applied every 2 weeks for 12 months. Two studies explored the combination of microneedling and TCA [105,106]. Intriguingly, a better response rate was observed with the application of 70% TCA than with 100% TCA. The reported adverse reactions included pain, erythema, post-inflammatory hyperpigmentation, infection, and scarring. Additional research is required to elucidate the role of TCA in the treatment of vitiligo, either as a monotherapy or as an adjuvant, and to determine the optimal concentrations of TCA for treating vitiligo, based on the affected area’s location.

## 12. Pseudocatalase

Oxidative stress in vitiligo, particularly linked to hydrogen peroxide (H_2_O_2_)-induced lipid peroxidation, is considered a potential mechanism underlying the development of the disease [107]. This phenomenon has been observed in vivo through direct quantification of H_2_O_2_ concentrations within depigmented epidermal layer [108]. Artificial catalysts capable of converting H_2_O_2_ into O_2_ and H_2_O have been proposed to address this oxidative imbalance.

One such active catalyst is a NB-UVB-activated bis-MnII(EDTA)2(HCO_3_^−^)_2_ complex (EDTA: ethylenediaminetetraacetate), commonly referred to as “pseudocatalase PC-KUS”. The largest study involving this treatment was conducted by Schallreuter et al. [109]. Their research involved 71 patients with generalized vitiligo divided into two subsets: a control group of 10 patients who underwent NB-UVB irradiation and 61 individuals who additionally received daily topical application of pseudocatalase. The study reported that progression was halted in 99% of patients treated with low-dose PC-KUS, in contrast to 30% in the control group. Moreover, repigmentation rates exceeding 75% were achieved in most body regions except for acral areas, and the outcomes exhibited statistically significant disparities compared with the control group.

These results initially appeared promising; however, subsequent clinical investigations carried out by Patel et al. [110], Bakis-Petsoglou et al. [111], Alshiyab et al. [112], and others found that pseudocatalase was no more effective than placebo creams or failed to demonstrate therapeutic benefit when combined with other treatments. In summary, despite initial optimism, pseudocatalase has not proven to be effective in the treatment of vitiligo, as validated by several clinical trials.

## 13. Prostaglandins

Latanoprost, an analog of prostaglandin F2 alpha (PGF2α), and bimatoprost, a prostamide analog, are primarily used in the treatment of glaucoma. Both have been documented to cause periocular skin hyperpigmentation as a side effect [113].

Prota et al. reported in a preclinical study that latanoprost upregulated TYR expression and activity, enhancing melanogenesis in melanocytes [114]. Furthermore, a case-control study revealed PGF2α levels in vitiligo-affected skin were higher than those in healthy skin, suggesting a potential role of PGF2α in the pathogenesis of vitiligo [115]. Subsequently, clinical studies have demonstrated the effectiveness of the topical application of latanoprost and bimatoprost in combination with phototherapy and microneedling [116,117,118,119].

Neinaa et al. [120] compared the efficacy of two different types of prostaglandins, prostaglandin E2 (PGE2) and PGF2α in the treatment of stable vitiligo, with NB-UVB phototherapy as an adjunct. Group 1 received intradermal injections of PGE2, Group 2 received PGF2α, and Group 3 received normal saline as a placebo once a week for 12 weeks. All groups received NB-UVB phototherapy twice a week for 3 months. The research findings revealed that there was no significant difference between the efficacy of PGE2 and PGF2α when combined with NB-UVB phototherapy; however, both were significantly more effective than NB-UVB alone. This suggests that the intradermal injection of PGF2α and PGE2 can be regarded as a simple and cost-effective technique for the treatment of stable vitiligo, with a relatively high patient satisfaction rate. However, the exact mechanism through which PGF2α enhances repigmentation in vitiligo remains unclear and warrants further investigation.

## 14. Considerations for the Future

The immunopathogenesis of vitiligo remains complex and is not fully understood, posing a significant challenge for its treatment. Recent research findings have identified multiple immune mechanisms and signaling pathways in the pathogenesis of vitiligo, including the JAK/STAT signaling pathway, the IFN-γ-CXCL9/10-CXCR3 pathway, Tregs, and tissue-resident memory T cells. Ideal treatments for vitiligo must address issues such as melanocyte proliferation, the inhibition of autoimmune damage, and the prevention of depigmentation recurrence. JAK inhibitors, for instance, have shown the ability to reduce CXCL9 and CXCL10 levels, effectively controlling vitiligo progression. Topical ruxolitinib has been approved for use in the U.S. and Europe, and oral upadacitinib has shown good efficacy in phase 2 clinical trials.

Combination therapy is likely to remain the most promising approach for vitiligo treatment in the future. Traditional therapies—such as hormone therapy, topical medications, phototherapy, and surgical treatment—continue to play a crucial role. However, JAK inhibitors currently face the challenge of re-pigmentation loss in the repigmented areas of vitiligo lesions, which may be related to the fact that JAK inhibitors can only block T cell recruitment and suppress the function of TRM in skin lesions but cannot reduce their numbers or eliminate them entirely [121]. Consequently, discontinuation of treatment may lead to disease progression, highlighting the need for durable solutions. PDE-4 inhibitors offer potential in this regard by preventing TRM enrichment in the epidermis.

Furthermore, phototherapy can induce apoptosis of T lymphocytes, downregulate inflammatory cytokines, upregulate interleukin-10, reduce the number of intraepithelial Langerhans cells, and induce tyrosinase activity [122]. These effects lead to increased melanogenesis, the proliferation of melanocytes, and their migration from the epidermal hair follicles, ultimately promoting repigmentation in the affected areas. In view of this, conducting in-depth exploration of combination therapies is highly promising, especially the potential synergistic effects among emerging therapies. For example, the combination of JAK inhibitors with phototherapy, or the combination of JAK inhibitors and PDE-4 inhibitors.

It is also essential to emphasize the need for translational research on the skin of people of color. The advantage of the skin of people of color is that there are more melanosomes in their melanocyte pool than in Caucasian skin, and targeted therapy may achieve better results. Despite the growing understanding of pathogenic pathways for vitiligo, many novel treatment approaches remain confined to in vitro or ex vivo studies, with limited clinical data available. As described in this article, treating vitiligo remains challenging. New treatment methods bring hope for more targeted and effective treatments; however, the efficacy and long-term safety of related drugs still require more large-sample, multi-center, randomized, double-blind, controlled clinical trials to verify and determine the most optimal drug regimens and dosage forms. Additionally, further investigation is required to establish strategies for maintaining treatment efficacy, determining maintenance doses, and preventing recurrence.

## 15. Conclusions

Although significant progress has been made in understanding the pathogenesis of vitiligo, many challenges remain to be addressed. Emerging treatment methods offer hope for more targeted and effective interventions; however, continuous research is crucial to fully realize the potential of these methods and improve the prognosis for patients with vitiligo.

## Figures and Tables

**Table 1:** Trials of emerging JAK inhibitors in vitiligo.

Drug	NCT/Country	Study Design	Outcome	Side Effects
Ruxolitinib JAK1/2 inhibitor	NCT04896385 The USA and Canada Trial phase: 2	Subjects: 60. Treatment period: 24 weeks. Randomized, double-blind. Group1: 1.5% ruxolitinib cream twice a day, Group2: vehicle.	Completed; no results published.	Not available.
	NCT02809976 The USA Trial phase: 2	Subjects: 11. Treatment period: 20 weeks. Single group, open-label. Group 1: ruxolitinib 1.5% phosphate cream twice daily.	Completed; four patients presented significant facial improvement, 23% of patients decreased VASI.	Only mild side effects.
	NCT03099304 The United Kingdom Trial phase: 2	Subjects: 157. Treatment period: 24 weeks. Randomized, double-blind. Group 1: ruxolitinib cream 1.5% twice daily, Group 2: ruxolitinib cream 1.5% once daily, Group 3: ruxolitinib cream 0.5% once daily, Group 4: ruxolitinib 0.15% once daily, Group 5: vehicle.	Completed; more patients in cream 1.5% twice daily, 1.5% once daily, 0.5% once-daily groups achieved F-VASI50 than the control groups. Extended treatment to 104 weeks: 54.9% (39/71) achieved F-VASI75, 50.0% (53/106) achieved T-VASI50.	All treatment-related adverse events were mild or moderate in severity.
	NCT05247489 The USA Trial phase: 2	Subjects: 55. Treatment period: 48 weeks. Randomized, open-label. Group 1: 1.5% ruxolitinib cream + narrow-band ultraviolet B phototherapy (NB-UVB), Group 2: 1.5% ruxolitinib cream monotherapy.	Completed; no results published.	Not available.
	NCT05750823 The USA Trial phase: 2	Subjects: 49. Treatment period: 48 weeks. Single group, open-label. Group 1: non-segmental vitiligo, apply ruxolitinib 1.5% cream twice a day to all depigmented areas.	Active, not recruiting; no results published.	Not available.
Tofacitinib JAK1/3 inhibitor	NCT: not available ([19]) The USA Trial phase: 2	Subjects: 16. Treatment period: mean time of 153 days (63–367). Single group, open-label. Group 1: Topical use of 2% tofacitinib twice daily.	Thirteen experienced repigmentation with four patients experiencing > 90% repigmentation, five patients experiencing 25–75% repigmentation, and four patients experiencing 5–15% repigmentation.	Only mild side effects.
	NCT05293119 The USA Trial phase: 1	Subjects: 80. Treatment period: 12 weeks. Randomized, open-label. Group 1: will receive 5mg Tofacitinib (oral), Group 2: will receive topical 0.1%Mometasone furoate.	Not yet recruiting	Not available.
Baricitinib JAK1/2 inhibitor	NCT04822584 France Trial phase: 2	Subjects: 49. Treatment period: 48 weeks. Randomized, open-label. Group 1: baricitinib 4 mg/day + narrowband UVB TL01 arm, Group 2: placebo.	Completed. The mean change in total VASI at week 36 was −44.8% (95% CI, −58.4% to −31.3%) for the baricitinib group and −9.2% (95% CI, −27.7% to 24.7%) for the placebo group.	One participant (5%) experienced back pain and one (5%) experienced a pulmonary embolism in the baricitinib group.
Upadacitinib JAK 1 inhibitor	NCT06118411 The USA Trial phase: 3	Subjects: 614. In Studies 1 and 2: Period A, participants take daily upadacitinib or placebo tablets for 48 weeks. Period B, 15 mg upadacitinib tablets daily for 112 weeks. In Study 3, participants get upadacitinib alone or with NB-UBV phototherapy for ≥24 weeks, then upadacitinib only.	Active, not recruiting.	Not available.
	NCT04927975 The USA Trial phase: 2	Subjects: 185. Treatment period: 24 weeks. Participants were randomized to 6-, 11-, or 22-mg/day upadacitinib or placebo.	Completed. Treatment with a 22 mg dose of upadacitinib led to a greater proportion of patients reporting less conspicuous vitiligo lesions compared to placebo (11.6% vs. 0%; *p* < 0.05).	Not available.
Ritlecitinib JAK 3 and TEC inhibitor	NCT05583526 The USA Trial phase: 3	Subjects: 581. Treatment period: 52 weeks. Randomized, double-blind. Group 1: ritilecitinib 50 mg QD arm, Group 2: placebo arm. Out of approximately 200 participants,	Active, not recruiting.	Not available.
	NCT03715829 The USA Trial phase: 2	187 patients subsequently received ritlecitinib at 200/50 mg daily in a 24-week extension period. Phase 2b, randomized, double-blind, placebo-controlled, parallel-group, multicenter, and dose-ranging study of 364 patients with face and body vitiligo treated for a 24-week dose-ranging period and 24-week extension period.	Completed. Significant differences from placebo in percent change from baseline in Facial-Vitiligo Area Scoring Index were observed for the 50 mg ritlecitinib groups with (−21.2 vs. 2.1; *p* < 0.001) or without (−18.5 vs. 2.1; *p* < 0.001) a loading dose and 30 mg ritlecitinib group (−14.6 vs. 2.1; *p* = 0.01). Accelerated improvement was observed after treatment with 200/50 mg ritlecitinib in the extension period (n = 187).	Only mild side effects.
Povorcitinib JAK1 inhibitor	NCT04818346 The USA Trial phase: 2	Subjects: 171. Treatment period: 24-week placebo-controlled double-blind treatment period, followed by a 28-week double-blind extension period; 171 patients were randomized to receive 15, 45, or 75 mg povorcitinib or placebo for 24 weeks, followed by extension 28 weeks.	Completed; no results published.	Not available.
	NCT06113471 The USA Trial phase: 3	Subjects: 444. Treatment period: 52 weeks. Randomized, double-blind. Povorcitinib or placebo (dose information not available).	Recruiting.	Not available.
	NCT06113445 The USA Trial phase: 3	Subjects: 444. Treatment period: 52 weeks. Randomized, double-blind. Povorcitinib or placebo (dose information not available).	Recruiting.	Not available.
Cerdulatinib SYK and JAK inhibitor (without JAK2)	NCT04103060 The USA Trial phase: 2a	Subjects: 33. Treatment period: 6 weeks. Randomized, double-blind. Group 1: Cerdulatinib 0.37% gel applied topically twice daily. Group 2: Vehicle gel applied topically twice daily.	Completed; no results published.	Not available.

## Data Availability

The original contributions presented in this study are included in the article, further inquiries can be directed to the corresponding author/s.

## Abbreviations

The following abbreviations are used in this manuscript:

cAMP	Cyclic adenosine monophosphate
CTLA-4	Cytotoxic T-lymphocyte-associated antigen-4
DCs	Dendritic cells
HSP	Heat shock protein
IFN	Interferon
IL	Interleukin
JAK	Janus kinase
mTOR	Mammalian target of rapamycin
PDE-4	Phosphodiesterase-4
PGE2	Prostaglandin E2
PGF2α	Prostaglandin F2 alpha
STAT	Signal transducer and activator of transcription
TCA	Trichloroacetic acid
TNF-α	Tumor necrosis factor α
Tregs	Regulatory functions of regulatory T cells
TRMs	Tissue-resident memory T cells
TYK2	Tyrosine kinase 2
TYR	Tyrosinase
U.S. FDA	United States Food and Drug Administration
VASI	Vitiligo Area Scoring Index
α-MSH	α-melanocyte-stimulating hormone
